# Digital screen time and speech-language delay in children: A cross-sectional study

**DOI:** 10.6026/973206300214956

**Published:** 2025-12-15

**Authors:** Priyasha Tripathi, Deepak K Uikey, Priyanka Verma

**Affiliations:** 1 Department of pediatrics, Atal Bihari Vajpayee govt medical College, Vidisha, Madhya Pradesh, India; 2 Department of ENT, Atal Bihari Vajpayee govt medical College Vidisha, Madhya Pradesh, India

**Keywords:** Digital screen time, speech delay, language development, children, parental supervision

## Abstract

Digital device use in early childhood has raised concerns regarding its impact on speech and language development. Hence, this cross- sectional study of 2000 children aged 6 months to 5 years examined the association between screen time and isolated speech delay. Overall, 60% showed speech delay, with the highest prevalence in toddlers (70%). Excessive screen time, poor-quality content and lack of parental supervision were significantly associated with speech delay across all age groups (p < 0.05). Thus, we show the need for regulated screen exposure, parental involvement and early intervention to support healthy language development.

## Background:

In today's digital era, smart devices have become ubiquitous in households worldwide, fundamentally altering how children learn, interact and communicate [1]. The American Academy of Pediatrics reports that children under 8 years spend an average of 2 hours and 19 minutes daily with screen media, a figure that has steadily increased over the past decade [2]. While digital media offers educational advantages, particularly during the COVID-19 pandemic when remote learning became necessary [3], prolonged unsupervised screen time during formative years can negatively impact language development [4]. Early speech development depends critically on responsive verbal interactions and rich environmental stimuli [5]. The first three years of life represent a period of remarkable neuroplasticity, during which foundational language skills are established through social interactions with caregivers [6]. Passive screen exposure reduces face-to-face conversation opportunities, potentially impairing both expressive and receptive language growth [7]. Several studies have demonstrated that excessive screen time is associated with delayed language acquisition, reduced vocabulary and poorer communication skills in young children [8, 9]. Recent research has highlighted concerning trends in digital parenting practices. Madigan *et al.* (2020) conducted a systematic review and meta-analysis showing that each additional hour of screen time in preschoolers correlated with a decrease in expressive language abilities [10,11-12]. The American Academy of Pediatrics (AAP) recommends zero screen exposure for children under 18 months (except for video calls) and a maximum of one hour of high-quality programming for children aged 2 to 5 years [13]. The Indian Academy of Pediatrics (IAP) has issued even stricter guidelines, recommending no screen time for children under 2 years and limiting screen time to a maximum of 1 hour daily for children aged 2-5 years [14]. Despite these clear recommendations, real-world implementation falls short, with many children exposed to significantly more screen time than advised [15]. While substantial research exists on this topic from Western countries, there is a paucity of data from Indian populations, where cultural context, socioeconomic factors and parenting practices may differ significantly [16]. Additionally, most studies have focused primarily on screen duration rather than examining the combined effects of content quality, device type, supervision patterns and contextual factors such as mealtime and bedtime screen use [17]. Therefore, it is of interest to address these gaps by examining the correlation between various aspects of screen exposure (duration, content quality, supervision patterns and contextual use) and isolated speech delay in a large cohort of Indian children aged 6 months to 5 years.

## Materials and Methods:

## Study design and setting:

A cross-sectional observational study was conducted between January 2022 and December 2022 at the Outpatient Department (OPD) and Inpatient Department (IPD) of the Pediatrics Department, Atal Bihari Vajpayee Government Medical College (ABVGMC), Vidisha, India. The study was approved by the Institutional Review Committee (IRC)/Institutional Ethics Committee (IEC) (approval number: ABVGMC/IEC/2021/ 42).

## Sample size and sampling technique:

A progressive sampling technique was employed to recruit participants. The sample size was calculated using the formula n = Z^2^pq/d^2^, where Z is the standard normal deviate (1.96 at 95% confidence level), p is the expected prevalence of speech delay (50% for maximum variability), q is 1-p and d is the margin of error (5%). This calculation yielded a minimum sample size of 384. To ensure adequate representation across age groups and to account for potential dropouts, a total of 2000 children were included in the study.

## Study population and grouping:

Children aged 6 months to 5 years attending the OPD and IPD for routine check-ups or minor illnesses were eligible for inclusion. Participants were divided into three age groups:

[1] Group 1: Infants (6 months to 1 year), n=500.

[2] Group 2: Toddlers (1-3 years), n=800.

[3] Group 3: Preschoolers (3-5 years), n=700.

##  Inclusion and exclusion criteria:

## Inclusion criteria:

[1] Children aged 6 months to 5 years.

[2] Parental consent for participation.

[3] Availability of at least one parent for interview and questionnaire completion.

## Exclusion criteria:

[1] Children with known genetic syndromes (e.g., Down syndrome, Fragile X syndrome).

[2] Children with diagnosed neurodevelopmental disorders (e.g., autism spectrum disorder, cerebral palsy).

[3] Children with significant hearing impairment (as confirmed by otoacoustic emissions or brainstem evoked response audiometry).

[4] Children with documented neurological disorders or traumatic brain injury.

[5] Children from multilingual households where more than two languages were spoken regularly.

## Data collection tools and procedures:

Data collection was performed by trained pediatricians and speech-language pathologists using the following tools: Structured Parental Questionnaire: A comprehensive questionnaire was administered to parents to collect information on:

[1] Demographic details (parental education, occupation, socioeconomic status).

[2] Screen time duration (hours per day).

[3] Type of screen devices used (mobile phones, tablets, television, computers).

[4] Content quality (educational, entertainment, age-appropriate).

[5] Supervision patterns (co-viewing with adults, solitary viewing).

[6] Contextual use of screens (during meals, bedtime, etc.).

[7] Behavioral patterns related to screen use.

The "Seven in Seven Screen Exposure Questionnaire," a validated tool for assessing screen time in young children, was incorporated into this questionnaire.

[1] Receptive-Expressive Emergent Language Test, Fourth Edition (REEL-4): This standardized assessment tool was used to evaluate speech and language development in children. The REEL-4 provides age-based norms for receptive and expressive language skills and helps identify language delays.

[2] Articulation Tests: Age-appropriate articulation assessments were conducted to evaluate speech sound production and identify articulation disorders.

[3] Structured Interviews: In-depth interviews were conducted with parents to gather qualitative information about screen use patterns, content preferences and observed behavioral changes in their children.

## Assessment of screen time guidelines:

Screen time practices were compared against established guidelines from the American Academy of Pediatrics (AAP) and the Indian Academy of Pediatrics (IAP):

AAP guidelines:

[1] For children <18 months: Avoid screen time except video chatting.

[2] For children 18-24 months: High-quality content only, co-viewed with caregiver.

[3] For children 2-5 years: Limit screen time to 1 hour/day of high-quality programming, co-viewed.

IAP guidelines (2021):

[1] No screen time for children under 2 years.

[2] Limit screen time to a maximum of 1 hour/day for children aged 2-5 years.

[3] Encourage screen-free meals, consistent sleep schedules and high parental interaction during media usage.

## Statistical analysis:

Data were analyzed using SPSS version 25.0 (IBM Corp., Armonk, NY, USA). Descriptive statistics were presented as frequencies, percentages, mean ±standard deviation (SD) and range as appropriate. Chi-square tests were applied to evaluate associations between categorical variables (screen time, supervision patterns, content quality) and speech delay. A p-value of <0.05 was considered statistically significant. The strength of association was measured using Cramer's V coefficient for chi-square tests.

## Results:

A total of 2000 children were included in the study, with a mean age of 2.9 ±1.4 years. Among these, 1200 children (60%) were identified with speech delay. The demographic characteristics of the study population are presented in Table 1 (see PDF). The distribution of screen time patterns and their association with speech delay across the three age groups are presented in Table 2 (see PDF). Behavioral issues observed in children with excessive screen time are presented in Table 3 (see PDF). Speech delay patterns varied significantly across age groups, with infants showing early signs related to limited vocalization. Among 500 infants, 280 (56%) demonstrated speech delay, primarily producing only monosyllabic sounds. Excessive screen exposure (>2 hours/day) was common in half of the infants, often on mobile devices and 30% lacked caregiver supervision. Sleep disturbances and poor feeding were reported in 12% of affected infants. A significant association between screen time and speech delay was confirmed (p < 0.05), with Cramer's V indicating a moderate strength of association (V = 0.32). Toddlers showed the highest vulnerability, with 560 out of 800 (70%) experiencing speech delays, often speaking only 2-3 unclear words and depending heavily on gestures. Excessive screen use (4-5 hours/day) was observed in 60% of toddlers and half lacked parental or teacher supervision. Behavioral problems such as aggression and temper tantrums were common, especially when screen access was restricted. Many toddlers also required screens for eating or sleeping. The correlation between screen exposure and speech delay was statistically significant (p < 0.05), with the strongest association across groups (Cramer's V = 0.42). Among 700 preschoolers, 360 (51.4%) had mild to moderate speech delay, with concerning behaviors such as imitating cartoon characters and using fictional names for family members. Although many preschoolers engaged in co-viewing, they were still exposed to inappropriate content like reels and irrelevant videos. Poor sleep habits were widespread. Statistical analysis confirmed a significant association between screen time and speech delay in this group (p < 0.05), with Cramer's V showing a moderate relationship (V = 0.28). Across all ages, lack of parental supervision (V = 0.48), excessive screen duration (V = 0.38) and poor content quality (V = 0.35) were the strongest predictors of speech delay.

## Discussion:

This study revealed a strong association between prolonged, unsupervised digital screen exposure and speech delay across all age groups, with toddlers (1-3 years) being the most affected. The findings align with previous research suggesting that early childhood is a critical period for language development, during which excessive screen time may interfere with essential social interactions and language learning opportunities [18]. The high prevalence of speech delay (60%) in our study cohort is concerning, particularly when compared to global estimates of 5-8% for preschool children [19]. This discrepancy may be attributed to several factors, including the high rates of excessive screen time (51.5% of children exposed to >2 hours daily), lack of parental supervision (32.5%) and exposure to poor-quality content (50%) observed in our study population. Additionally, the limited parental awareness of screen time guidelines (only 20% were aware) and inability to identify speech delay (60% of parents) likely contributed to the high prevalence [20, 21]. This found that excessive screen time reduces expressive language capabilities. The toddler group in our study showed the highest prevalence of speech delay (70%), consistent with research indicating that children aged 1-3 years are particularly vulnerable to the effects of screen time due to the rapid language development occurring during this period [22,23]. The impact of content quality on language development emerged as a significant factor in our study. Children exposed to age-inappropriate or poor-quality content showed higher rates of speech delay and were more likely to use inappropriate language or mimic cartoon characters rather than developing age-appropriate communication skills. Our observation of children referring to caregivers using cartoon character names rather than proper names suggests that excessive exposure to such content may interfere with social referencing and real-world communication skills. The role of parental supervision in mitigating the negative effects of screen time was evident in our findings. Children with unsupervised screen exposure showed significantly higher rates of speech delay compared to those with co-viewing experiences. However, even co-viewing posed risks when inappropriate content was involved, highlighting the dual challenge of limiting screen exposure while ensuring quality supervision. This finding supports the recommendations of both the American Academy of Pediatrics and the Indian Academy of Pediatrics, which emphasize the importance of high-quality, co-viewed content for young children [24, 25]. Behavioral issues associated with excessive screen time were prominent in our study, particularly in the toddler group. Sleep disturbances, feeding difficulties, aggression and temper tantrums were significantly more common in children with excessive screen exposure. These findings are consistent with research by Al Hosani *et al.* [26], who reported that toddlers with over 3 hours of screen time showed a 40% higher risk of behavioral problems, even when controlling for parental education and socioeconomic background. The socioeconomic factors identified in our study provide important context for understanding screen time patterns. The high proportion of parents with limited education (40% of mothers with education up to 5th standard, 50% of fathers with education up to 10th standard) and working in semi-skilled occupations (60%) may contribute to both increased screen time use and limited awareness of its potential impacts. This highlights the need for targeted interventions in resource-limited settings where mobile screens often replace toys, books and direct caregiver interaction [27]. Our study adds to the limited data from Indian populations on this topic, addressing a significant research gap. While the findings are generally consistent with international research, the higher prevalence of speech delay and specific behavioral patterns observed in our cohort suggest that cultural and socioeconomic factors may influence how screen time impacts child development in the Indian context [28]. Several limitations of our study should be acknowledged. The cross-sectional design precludes establishing causality between screen time and speech delay. Additionally, the study was conducted in a single medical center, which may limit the generalizability of findings. Parental reporting of screen time and child behaviors may be subject to recall bias and social desirability effects. Future longitudinal studies with objective measures of screen time and more diverse populations would strengthen the evidence base. The implications of our findings for clinical practice and public health are significant. Pediatric healthcare providers should routinely screen for excessive screen time and provide anticipatory guidance to parents about appropriate screen use. Parent education programs should emphasize the importance of high-quality content, co-viewing and establishing screen-free zones and times, particularly during meals and before bedtime. Early identification and intervention for speech delay are crucial, as timely support can significantly improve outcomes [29].

## Conclusion:

Excessive screen time is significantly associated with isolated speech delay in young children, with toddlers being the most vulnerable group. Screen content, duration, and parental co-viewing strongly influence speech and behavioral outcomes. Promoting balanced screen use, parental awareness, and early intervention is essential to support healthy speech and language development.
